# Distal Femur Morphology in a Brazilian Population: Challenging the Universal Use of 3° of External Rotation

**DOI:** 10.1055/s-0044-1800923

**Published:** 2025-03-12

**Authors:** Enzo Mameri, Isabelle Kaptzky Ballarini, Maria Beatriz Pinheiro Leonel, Marcio de Castro Ferreira, Marcus Vinicius Malheiros Luzo, Marcelo Seiji Kubota

**Affiliations:** 1 Departamento de Ortopedia e Traumatologia, Escola Paulista de Medicina, Universidade Federal de São Paulo, São Paulo, SP, Brasil

**Keywords:** arthroplasty, replacement, knee, knee joint, magnetic resonance imaging, rotation

## Abstract

**Objective** To evaluate the external rotation angle between the transepicondylar axis (TEA) and the posterior condylar axis (PCA) in a Brazilian population to determine whether the universal use of 3° of external rotation of the femoral component is appropriate in these patients undergoing total knee arthroplasty (TKA).

**Methods** We measured the angle of external rotation in 167 magnetic resonance imaging (MRI) scans using 4 different methods for the PCA according to the posterior landmarks: measurement A – chondral prominences; measurement B – bony prominences; measurement C – lateral bony prominence and medial chondral prominence; and measurement D – lateral chondral prominence and medial bony prominence. We statistically compared these measurements with the traditional 3° of external rotation used in TKA.

**Results** The mean angles of external rotation in measurements A, B, C, and D were of 5.44° ± 2.39°, 4.94° ± 2.10°, 8.56° ± 2°, and 2.33° ± 2° respectively. All measurement methods resulted in significant differences regarding the universal value of 3° ( *p* > 0.0001). All intergroup comparisons showed statistical differences among the measurement methods ( *p* > 0.0001), except for the comparison between measurements A and B ( *p* = 0.1614).

**Conclusion** Using a chondral or bony reference point for the PCA, the TEA in the Brazilian population studied presented approximately 5° of external rotation. The traditional rotation of the femoral component of 3° regarding the PCA may be insufficient in the Brazilian population, especially in valgus knees. Therefore, we emphasize the significance of an individualized approach to achieve the ideal rotational alignment of TKA components.

## Introduction

Total knee arthroplasty (TKA) is the gold-standard surgical procedure for advanced tricompartmental knee osteoarthritis, with satisfactory pain and function improvement. 1 Multiple factors determine the survival and successful functional outcomes of TKA, including correct component alignment. 1 2 3 4 5

In the axial plane, rotational alignment of the femoral component greatly influences postoperative knee kinematics, particularly patellar tracking. To define femoral rotational alignment, several anatomical parameters are discussed, including the transepicondylar axis (TEE), the trochlear groove line (Whiteside line), the posterior condylar axis (PCA) or, even, according to the balanced extension gap. 2 In an arthroplasty following the principle of mechanical alignment based on measured resection, the literature widely accepts the femoral component positioning parallel to the surgical TEA. 3 4 5 Therefore, to achieve the final parallelism of the TEA component, the standard instrumentation of commercially-available prostheses is typically designed for a section at 3° of external rotation regarding the PCA, since the femoral condylar posterior surface supports the guide.

However, the bone morphology of the distal third of the femur may differ in populations or ethnic groups, as previously reported in Eastern populations. 5 6 7 8 With this context in mind, it is possible that the universal application of 3° of external rotation regarding the PCA is not adequate for all populational groups.

The main objective of the present study was to evaluate the angular relationship between the femoral TEA and femoral PCA in a Brazilian population to identify whether the empirical application of 3° of external rotation of the femoral component is appropriate for this population.

## Materials and Methods

### Sample and eligibility criteria

We retrospectively analyzed magnetic resonance imaging (MRI) scans from the Radiology Department of a quaternary hospital after approval by the Institutional Ethics Committee. The study included previously-collected images of patients older than 60 years of age (a clinically-relevant age range for TKA indication) complaining of knee pain and without osteoarthritis greater than grade 2 on the Kellgren-Lawrence classification or intra- or extra-articular deformities. We excluded patients with a history of lower limb surgery, fractures, or pre-existing systemic inflammatory disease.

Sample size determination considered a 5% significance level. The correlation between the analyzed variables was deemed moderate, and the statistical test power was of 80%. Thus, the minimum number of patients was of 55 for each sex, considering the significant anthropometric differences between male and female subjects.

### Radiological analysis

**Fig. 1:**
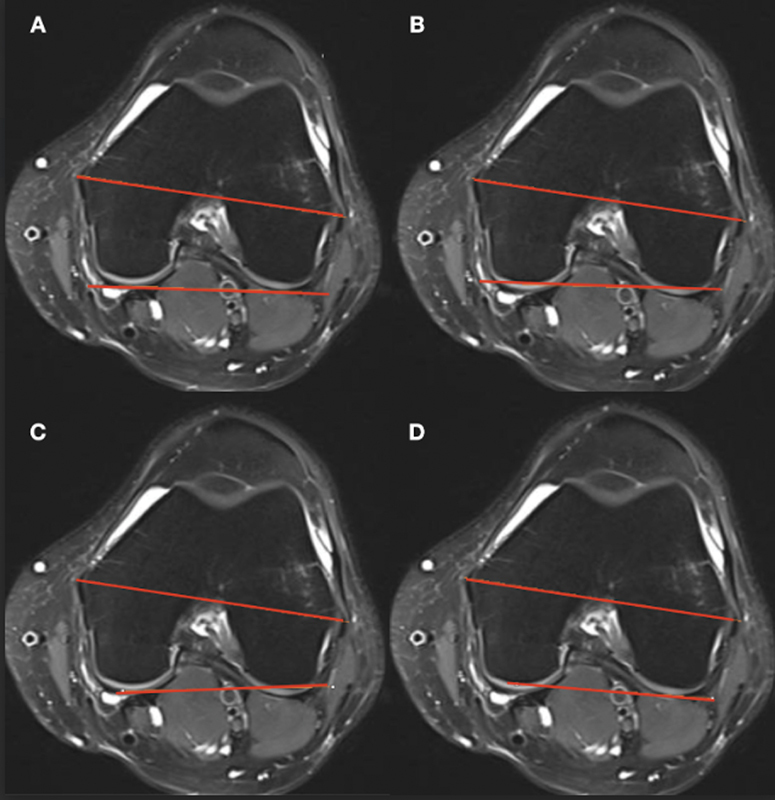
Measurement of the posterior condylar axis (PCA) according to several anatomical references. ( **A** ) PCA defined by the most posterior chondral prominences of the medial and lateral femoral condyles; ( **B** ) PCA defined by the most posterior bony prominences of the medial and lateral femoral condyles; ( **C** ) PCA defined by the medial femoral condylar chondral prominence and lateral femoral condylar bony prominence; and ( **D** ) PCA defined by the medial femoral condylar bony prominence and lateral femoral condylar chondral prominence.

We analyzed axial T2-weighted MRI scans in a single section containing the topographies of the medial and lateral epicondyles and posterior femoral condyles. The TEA definition relied on the apices of the medial and lateral epicondylar prominences, and the PCA definition relied on a line tangent to the most posterior extension of the femoral condyles. The primary study outcome was the angular relationships between the TEA and the PCA. To identify the potential influence of the posterior condylar cartilage on PCA measurement, we used four methods with different anatomical landmarks:

- Measurement A – defided by the most posterior chondral prominences of the posterior medial and lateral femoral condyles ( Fig. 1A ).
- Measurement B – defided by the most posterior bony prominences of the posterior medial and lateral femoral condyles ( Fig. 1B ).
- Measurement C – defided by the medial femoral condylar chondral prominence and lateral femoral condylar bony prominence ( Fig. 1C ).
- Measurement D – defided by the medial femoral condylar bony prominence and lateral femoral condylar chondral prominence ( Fig. 1D ).

The same orthopedist performed all angular measurements using a specific software (Synapse Radiology PACS, Fujifilm, Minato, Tokyo, Japan).

### Statistical analysis

To assess whether the angular measurements of external rotation between the TEA and the PCA presented statistically significant differences from the universal value of 3° of external rotation used in traditional TKA instrumentation, we used the Student's *t* -test for a single sample, which is suitable for comparing means between a sample and an established target value. The significance level was of 5% ( *p* < 0.05).

We performed analysis of variance (ANOVA) to detect whether there was any difference between the different forms of rotation of the TEA regarding the PCA. When the ANOVA revealed statistically significant differences, the Tukey test was used for post-hoc comparisons between the groups to identify actual differences comparing each pair of measurements (A-B, A-C, A-D, B-C etc.).

## Results

**Fig. 2:**
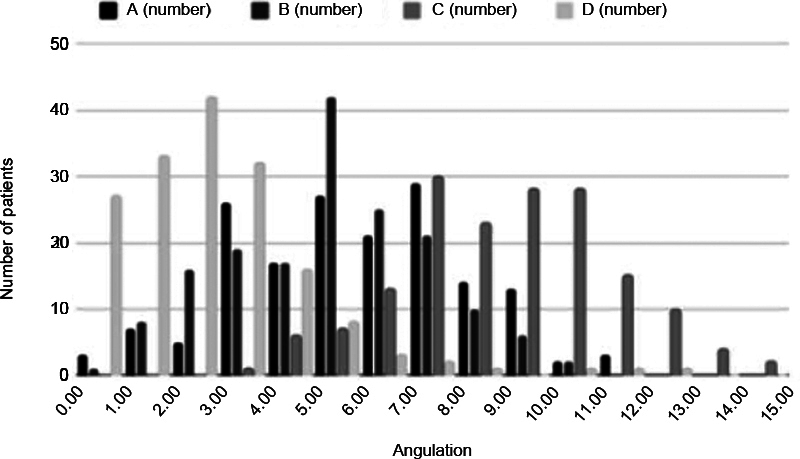
Graph of the distribution of the rotation angles of the transepicondylar axis regarding each posterior condylar axis measurement (A, B, C, and D).

The present study included 167 knees, 106 from female and 61 from male patients, with 95 left and 72 right knees. The mean age of the final sample was of 67.57 ± 6.22 (range|: 60–85) years. Figs. 2 3 4 show the distribution of the measurements for the total sample, and for the male and female patients respectively.

**Fig. 3:**
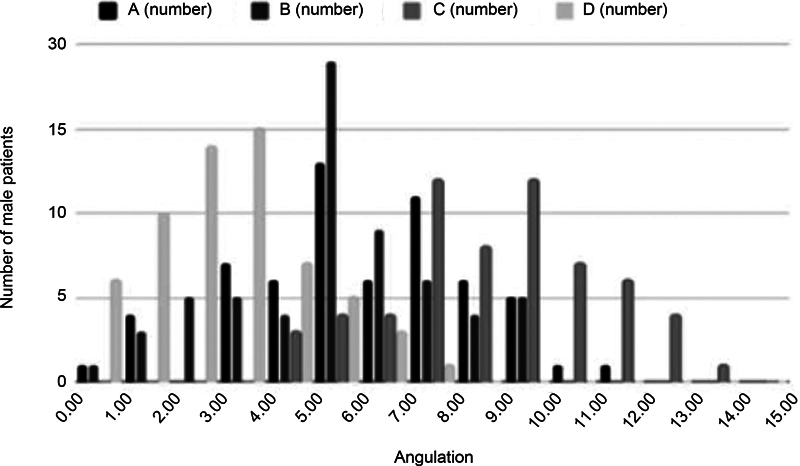
Graph of the distribution of the rotation angles of the transepicondylar axis regarding each posterior condylar axis measurement (A, B, C, and D) in male patients.

**Fig. 4:**
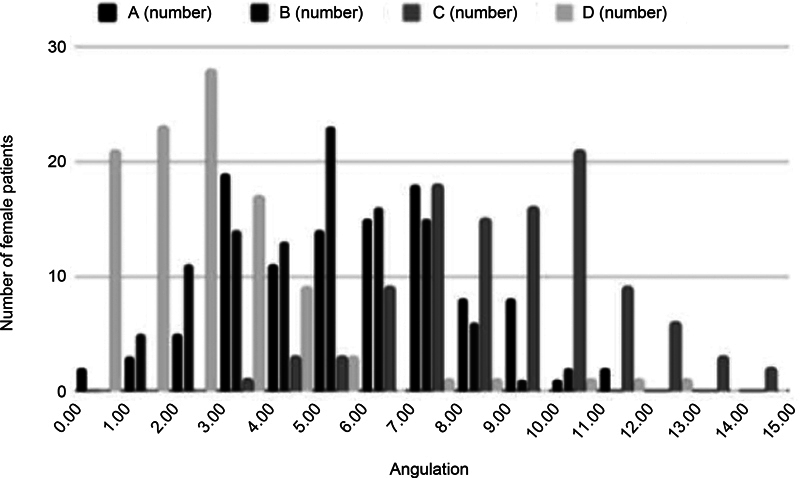
Graph of the distribution of the rotation angles of the transepicondylar axis regarding each posterior condylar axis measurement (A, B, C, and D) in female patients.

The mean angular values of external rotation of the TEA regarding the PCA, according to measurements A, B, C, and D, were of 5.44°, 4.94°, 8.57°, and 2.33° respectively. These values, in all forms of rotation measurement (A, B, C, D), presented statistically significant differences ( *p* < 0.0001) from the universal value of 3° of external rotation used in the standard TKA instrumentation ( Table 1 ). There was no statistically significant difference between male and female patients in any measurement (p > 0.05).

**Table 1:** Angular measurements of external rotation (ER) of the transepicondylar axis (TEA) regarding the posterior condylar axis (PCA) and statistical comparison with the universal value of 3° in total knee arthroplasty

	TEA-PCA external rotation: mean ± standard deviation	*p* versus universal value of 3° of external rotation
**Measurement A**		
Male sex	5.590° ± 2.404°	
Female sex	5.358° ± 2.391°	
Total	5.443° ± 2.384°	*p* < 0.0001
**Measurement B**		
Male sex	5.115° ± 2.119°	
Female sex	4.846° ± 2.051°	
Total	4.946° ± 2.097°	*p* < 0.0001
**Measurement C**		
Male sex	8.334° ± 2.190°	
Female sex	8.698° ± 2.132°	
Total	8.347° ± 2.273°	*p* < 0.0001
**Measurement D**		
Male sex	2.639° ± 1.684°	
Female sex	2.263° ± 2.213°	
Total	2.335° ± 2.002°	*p* < 0.0001

Among the different measurement methods, based on combinations of bone or chondral limits of the condyles, ANOVA revealed statistically significant differences between the groups ( *p* < 0.0001), with a large effect size (f = 1.02). Next, we compared each group using the Tukey's (Honest Significant Difference, HSD) test. This test detected statistically significant differences ( *p* < 0.0001) for all measurement pairs (A-C, A-D, B-C, B-D, C-D), except for A and B ( *p* = 0.161; Table 2 ).

**Table 2:** Statistical intergroup comparison of different ways of measuring the external rotation of the transepicondylar axis regarding the posterior condylar axis

Comparison	Difference	Standard error	Confidence interval	*p* -value
**A-B**	0.497	0.1691	−0.119–1.13	0.1614
**A-C**	3.1257	0.1691	2.5097–3.7418	< 0.0001
**A-D**	3.1078	0.1691	2.4918–3.7238	< 0.0001
**B-C**	3.6228	0.1691	3.0067–4.2388	< 0.0001
**B-D**	2.6108	0.1691	1.9947–3.2268	< 0.0001

## Discussion

The most significant finding of the present study is the external rotation profile of the TEA regarding PCA specifically for a Brazilian population, which presents a significant difference to the universal value of 3°. Our results show that using the parameters previously established in the literature, such as 3° of external rotation for the axial positioning of the femoral component, may not be an accurate method for the Brazilian population.

In a pivotal study, Berger et al. 7 demonstrated, in the axial plane, the requirement of approximately 3° of external rotation of the femoral component regarding the plane of the posterior condyles as a landmark. However, the results from this American population do not necessarily reflect the reality of populations with different ethnic compositions. Using similar measurements, Murgier et al. 8 described angles of 6.4° of external rotation in the Asian population. In another study, Pun et al. 9 found 4.6° of rotation for the Indian population. Therefore, given the discrepancy in values presented in the literature according to the ethnic group studied, the surgeon should question whether using anatomical references based on other populations may interfere with the planning for their patients.

In the present study, when the reference for the measurements was the intact chondral and bicondylar bone structures (groups A and B), there were no significant differences between the mean angles of 5.44° and 4.94°. Given the conditions of equal posterior condylar wear, the chance of error in rotational positioning is lower if the surgeon applies an external rotation greater than that traditionally stipulated in the cutting guides, using an approximate value of 5° of external rotation empirically. The results of a previous study by Loures et al. 10 corroborate the need for higher external rotation of the femoral component in the Brazilian population, for they identified a mean value of 6.89°. However, the authors 10 analyzed the PCA with a single measurement.

Specifically regarding the rotational positioning of the femoral component using the PCA and considering the anatomical parameters of the lateral and medial chondral bony prominence of the posterior femoral condyles (group C), we found relevant results for the difference in mean measurements of 8.56°. Thus, one must pay attention to the chondral profile of the posterior femoral condyles for rotational planning of the femoral component when using the condylar support guide. It is worth noting that the findings of the present study may be valuable in lateral posterior chondral wear, which was more prevalent in knees with valgus overload and resembled measurement C in the current study. In this scenario, the approximate mean divergence would be an additional 5° of external rotation regarding the universal empirical value of 3°. This fact would result in the positioning of the femoral component in internal rotation, which would contribute to an increase in lateral patellofemoral conflict and increase retinacular tension and patellar tilt, conditions that favor post-arthroplasty patellofemoral syndrome. 11 12 13 14 The resulting non-physiological knee kinematics entails the potential for worse clinical outcomes and postoperative anterior pain. 3 4 5

Nam et al. 15 showed that the average thickness of the lateral posterior femoral condyle cartilage is greater than the cartilage thickness of the medial posterior femoral condyle, with an average difference of 0.4 mm. This discrepancy may justify the significant difference in the angular values between the different forms of PCA measurements. In addition, the inequality in the chondral thickness of the femoral condyles in knee osteoarthritis may vary according to limb axis deviations and contribute as a causal factor for the error in determining the PCA. 16 Tashiro et al. 17 and Yang et al. 18 , when comparing the chondral thickness in the posterior aspect of the lateral and medial condyles, demonstrated that a thinning of just 2 mm of the cartilage in the lateral condyle can generate divergences of approximately 2° in the rotational alignment of the femoral implant.

The results of the present study provide additional support for the recommendation to use more than one method to measure the rotational positioning of the femoral component to reduce potential alignment errors due to anatomical variations, in addition to potential interobserver differences when defining the anatomical parameters used as landmarks. 19 20

The current study has some limitations. Limbs with valgus may present hypoplasia of the lateral femoral condyle, and we did not analyze the mechanical axes of the lower limbs, introducing a potential bias. Another limitation is that the group of the Brazilian population studied, in terms of number and age, may not reflect anatomically the entire Brazilian population due to the great ethnic diversity found in Brazil.

## Conclusion

Considering exclusively chondral or exclusively bony posterior references, the TEA in the Brazilian population studied presents approximately 5° of external rotation regarding the PCA. Therefore, the empirical application of 3° of external rotation in the femoral component may be insufficient for adequate rotational alignment in the Brazilian population, which may be even more pronounced in genu valgum. Thus, it is critical to consider the need for individualized techniques for optimal rotational positioning of the femoral component to obtain better accuracy.
