# Supplementary material for: Distal Femur Morphology in a Brazilian Population: Challenging the Universal Use of 3° of External Rotation
Source: Rev Bras Ortop (Sao Paulo). 2025 Mar 12;60(1):s00441800923. doi: 10.1055/s-0044-1800923 (PMC11903119; doi:10.1055/s-0044-1800923)
Supplement: Related texts

# Research article: Morfologia do fêmur distal em uma população brasileira: Desafiando o uso universal de 3° de rotação externa

**Keywords:** articulação do joelho, artroplastia do joelho, imageamento por ressonância magnética, rotação

## Abstract

**Objetivo** Avaliar, em uma população brasileira, o ângulo de rotação externa entre o eixo transepicondilar (ETE) e o eixo condilar posterior (ECP), a fim de estipular se o uso universal de 3° de rotação externa do componente femoral é apropriado neste grupo de pacientes na artroplastia total do joelho (ATJ).

**Métodos** O ângulo de rotação externa foi medido em 167 imagens de ressonância magnéticas utilizando-se 4 métodos diferentes para o ECP, de acordo com os pontos de referências posteriores: medida A – proeminências condrais; medida B – proeminências ósseas; medida C – proeminências ósseas lateral e condral medial; e medida D – proeminências condral lateral e óssea medial. As medidas obtidas foram comparadas estatisticamente com os valores tradicionais de rotação externa de 3° empregados na ATJ.

**Resultados** As médias do ângulo de rotação externa para as medidas A,B,C,D foram de 5,44° ± 2,39°, 4,94° ± 2,10°, 8,56° ± 2° e 2,33° ± 2°, respectivamente. Todos os métodos de medição resultaram em diferenças significativas em relação ao valor universal de 3° ( *p* > 0,0001). Diferenças estatísticas foram encontradas em todas as comparações intergrupos entre os métodos de medição ( *p* > 0,0001), exceto na comparação entre as medidas A e B ( *p* = 0,1614).

**Conclusão** Utilizando um ponto de referência condral ou ósseo para o ECP, o ETE na população brasileira estudada apresentou aproximadamente 5° de rotação externa. A rotação tradicional do componente femoral de 3° em relação ao ECP pode ser insuficiente na população brasileira, sobretudo no joelho valgo. Assim, enfatizamos a importância de uma abordagem individualizada para alcançar o alinhamento rotacional ideal dos componentes na ATJ.

## Introdução

A artroplastia total do joelho (ATJ) é o procedimento cirúrgico considerado padrão-ouro para casos de osteoartrite tricompartimental avançada do joelho, com resultados satisfatórios em termos de melhora da dor e função. 1 A sobrevida e o sucesso dos desfechos funcionais de uma ATJ são determinados por múltiplos fatores, entre eles, o correto alinhamento dos componentes. 1 2 3 4 5

No plano axial, o alinhamento rotacional do componente femoral tem grande influência na cinemática pós-operatória do joelho, particularmente no trilhamento patelar. Para a defineção do alinhamento rotacional femoral, são discutidos diversos parâmetros anatômicos, entre eles, o eixo transepicondilar (ETE), a linha do sulco troclear (linha de Whiteside), o eixo condilar posterior (ECP) ou, ainda, de acordo com o gap de extensão balanceado. 2 Quando se realiza uma artroplastia seguindo o princípio de alinhamento mecânico baseado na ressecção medida, o posicionamento do componente femoral paralelo ao ETE cirúrgico é amplamente aceito na literatura como o ideal. 3 4 5 Para atingir o paralelismo final do componente ao ETE, portanto, o instrumental-padrão das próteses comercialmente disponíveis é tipicamente projetado para um corte a 3° de rotação externa em relação ao ECP, uma vez que o guia é apoiado na superfície posterior dos côndilos femorais.

No entanto, a morfologia óssea do terço distal do fêmur pode apresentar diferenças entre grupos populacionais ou étnicos, como publicado previamente em populações orientais. 5 6 7 8 Com esse contexto em mente, é possível que a aplicação universal dos 3° de rotação externa em relação ao ECP não seja adequada para todos os grupos populacionais.

O objetivo principal deste estudo foi avaliar a relação angular entre o ETE e o ECP femorais em uma população brasileira, a fim de identificar se a aplicação empírica de 3° de rotação externa do componente femoral é apropriada para este grupo populacional.

## Materiais e Métodos

## Amostra e critérios de elegibilidade

Após aprovação do Comitê de Ética em Pesquisa (CEP) institucional, foram analisados retrospectivamente exames de ressonância magnética (RM) realizados no Departamento de Radiologia de um hospital quaternário. Foram incluídas imagens previamente coletadas de pacientes maiores de 60 anos (faixa etária clinicamente relevante para a indicação de ATJ) com queixa de dor no joelho, sem osteoartrite de grau maior do que 2 segundo a classificação de Kellgren-Lawrence, e sem deformidades intra ou extra-articulares. Foram excluídos pacientes com histórico de cirurgia nos membros inferiores, fraturas ou doença inflamatória sistêmica pré-existente.

Para a determinação do tamanho amostral, considerou-se nível de significância de 5%, a correlação entre as variáveis analisadas, como moderada, e poder do teste estatístico de 80%. Desta forma, o tamanho mínimo da amostra foi de 55 pacientes de cada sexo, considerando as diferenças antropométricas significativas entre os indivíduos do sexo masculino e feminino.

## Análise radiológica

Foram analisadas imagens axiais em sequência T2, no plano que continha em um único corte as topografias dos epicôndilos medial e lateral e dos côndilos femorais posteriores. O ETE foi definido pelos ápices das proeminências epicondilares medial e lateral, e o ECP, por uma linha que tangencia a extensão mais posterior dos côndilos femorais. A medida de desfecho primária do estudo corresponde às relações angulares medidas entre os ETEs e ECPs. A fim de identificar a influência que a cartilagem condilar posterior pode exercer na mensuração do ECP, este eixo foi medido de quatro maneiras a partir de referenciais anatômicos distintos:

- Medida A –definida pelas proeminências condrais mais posteriores dos côndilos femorais medial e lateral posteriores ( Fig. 1A ).
- Medida B – definida pelas proeminências ósseas mais posteriores dos côndilos femorais medial e lateral posteriores ( Fig. 1B ).
- Medida C – definida pela proeminência condral condilar femoral medial e proeminência óssea condilar femoral lateral ( Fig. 1C ).
- Medida D – definida pela proeminência óssea condilar femoral medial e proeminência condral condilar femoral lateral ( Fig. 1D ).

**Fig. 1:** Mensuração do eixo condilar posterior (ECP) conforme referenciais anatômicos distintos. ( **A** ) ECP definido pelas proeminências condrais mais posteriores dos côndilos femorais medial e lateral; ( **B** ) ECP definido pelas proeminências ósseas mais posteriores dos côndilos femorais medial e lateral; ( **C** ) ECP definido pela proeminência condral condilar femoral medial e proeminência óssea condilar femoral lateral; e ( **D** ) ECP definido pela proeminência óssea condilar femoral medial e pela proeminência condral condilar femoral lateral.

Todas as medidas angulares foram aferidas pelo mesmo ortopedista em um programa específico (Synapse Radiology PACS, Fujifilm, Minato, Tóquio, Japão).

## Análise estatística

A fim de avaliar se as medidas angulares de rotação externa entre o ETE e ECP encontradas diferiam estatisticamente do valor universal de 3° de rotação externa utilizado na instrumentação tradicional da ATJ, foi utilizado o teste *t* de Student para uma amostra única, próprio para a comparação de médias entre uma amostra e um valor-alvo estabelecido. O nível de significância adotado foi de 5% ( *p* < 0.05).

A análise da variância ( *analysis of variance* , ANOVA, em inglês) foi realizada para detectar se existia alguma diferença entre as distintas formas de rotação do ETE em relação ao ECP. Quando a ANOVA revelou diferenças estatisticamente significativas, foi então utilizado o teste de Tukey para comparações *post hoc* entre os grupos, de forma a identificar as diferenças verdadeiras ao se comparar cada par de medidas (A-B, A-C, A-D, B-C etc.).

## Resultados

Foram incluídos 167 joelhos: 106 de pacientes do sexo feminino e 61 de pacientes do sexo masculino, 95 joelhos esquerdos e 72 joelhos direitos. A média de idade da amostra final foi de 67,57 ± 6,22 (variação: 60–85) anos. As distribuições das medidas encontradas para a amostra total e, especificamente, para os pacientes dos sexos masculino e feminino, estão representada nas Figs. 2 3 4 , respectivamente.

**Fig. 2:** Gráfico da distribuição dos ângulos de rotação do eixo transepicondilar em relação a cada medida do eixo condilar posterior (A,B,C e D).

**Fig. 3:** Gráfico da distribuição dos ângulos de rotação do eixo transepicondilar em relação a cada medida do eixo condilar posterior (A,B,C e D) nos pacientes do sexo masculino.

**Fig. 4:** Gráfico da distribuição dos ângulos de rotação do eixo transepicondilar em relação a cada medida do eixo condilar posterior (A,B,C e D) nos pacientes do sexo feminino.

Os valores angulares médios de rotação externa do ETE em relação ao ECP, de acordo com as medidas A, B, C e D, foram de 5,44°, 4,94°, 8,57° e 2,33°, respectivamente. Os valores encontrados nesta amostra em todas as formas de medida da rotação (A, B, C, D) apresentam diferenças estatisticamente significativas ( *p* < 0,0001) com relação ao valor universal de 3° de rotação externa utilizado no instrumental-padrão de ATJ ( Tabela 1 ). Em nenhum dos tipos de medida foi encontrada diferença estatisticamente significativas entre pacientes dos sexos feminino e masculino ( *p* > 0,05).

**Tabela 1:** Medidas angulares da rotação externa (RE) do eixo transepicondilar (ETE) em relação ao eixo condilar posterior (ECP) e comparação estatística com o valor universal de 3° na artroplastia total do joelho

	Rotação externa ETE-ECP: média ± desvio padrão	*p* versus valor universal de 3° de rotação externa
**Medida A**		
Sexo masculino	5,590° ± 2,404°	
Sexo feminino	5,358° ± 2,391°	
Total	5,443° ± 2,384°	*p* < 0,0001
**Medida B**		
Sexo masculino	5,115° ± 2,119°	
Sexo feminino	4,846° ± 2,051°	
Total	4,946° ± 2,097°	*p* < 0,0001
**Medida C**		
Sexo masculino	8,334° ± 2,190°	
Sexo feminino	8,698° ± 2,132°	
Total	8,347° ± 2,273°	*p* < 0,0001
**Medida D**		
Sexo masculino	2,639° ± 1,684°	
Sexo feminino	2,263° ± 2,213°	
Total	2,335° ± 2,002°	*p* < 0,0001

Entre as diferentes formas de medida, baseadas nas combinações de limites ósseos ou condrais dos côndilos, a ANOVA revelou a presença de diferenças estatisticamente significativas entre os grupos ( *p* < 0,0001), com medida de efeito considerada grande (f = 1,02). Em seguida, foram realizadas comparações entre cada par de grupos por meio do teste de diferença significativa honesta (Honest Significant Difference, HSD, em inglês) de Tukey, em que foram encontradas diferenças estatisticamente significativas ( *p* < 0,0001) para todas os pares de medidas (A-C, A-D, B-C, B-D, C-D), exceto quando comparadas as medidas A e B ( *p* = 0,161; Tabela 2 ).

**Tabela 2:** Comparação estatística intergrupos das diferentes formas de medida da rotação externa do eixo transepicondilar em relação ao eixo condilar posterior

Comparação	Diferença	Erro padrão	Intervalo de confiança	Valor de *p*
**A-B**	0,497	0,1691	−0,119–1,13	0,1614
**A-C**	3,1257	0,1691	2,5097–3,7418	< 0,0001
**A-D**	3,1078	0,1691	2,4918–3,7238	< 0,0001
**B-C**	3,6228	0,1691	3,0067–4,2388	< 0,0001
**B-D**	2,6108	0,1691	1,9947–3,2268	< 0,0001

## Discussão

O achado mais importante do presente estudo é o perfil de rotação externa do ETE em relação ao ECP específico de uma população brasileira, que apresenta diferença significativa em relação à medida universal de 3°. De acordo com os resultados encontrados, a utilização de parâmetros preestabelecidos na literatura, como 3° de rotação externa para o posicionamento axial do componente femoral, pode não ser um método acurado na população brasileira.

Berger et al. 7 demonstraram, em um estudo divisor de águas, que no plano axial são necessários aproximadamente 3° de rotação externa do componente femoral em relação ao plano dos côndilos posteriores como referência. Os resultados encontrados nesta população americana, no entanto, não necessariamente refletem a realidade de populações de composição étnica distinta. Murgier et al. 8 descreveram, para a mesma mensuração, na população asiática, angulações de 6,4° de rotação externa. Em outro estudo, Pun et al. 9 encontraram resultados de 4,6° de rotação para a população indiana. Desta forma, diante da discrepância de valores apresentados na literatura conforme a etnia estudada, o cirurgião deve questionar se o uso de referências anatômicas pautadas em outras populações pode interferir no planejamento feito para seus pacientes.

Neste estudo, quando as medidas foram referenciadas pelas estruturas íntegras condrais e ósseas bicondilares (grupos A e B), não se observaram diferenças significativas entre os ângulos médios de 5,44° e 4,94°. Diante de condições de igualdade de desgastes condilares posteriores, portanto, a chance de erro no posicionamento rotacional é menor se aplicada rotação externa superior à tradicionalmente estipulada nos guias de corte, utilizando um valor aproximado de 5° de rotação externa empiricamente. Os resultados de um estudo conduzido por Loures et al. 10 corroboram a necessidade de maior rotação externa do componente femoral na população brasileira, pois identificou-se um valor médio de 6,89°. Os autores, 10 no entanto, analisaram o ECP com uma só medida.

Especificamente em relação ao posicionamento rotacional do componente femoral usando o ECP e considerando os parâmetros anatômicos da proeminência óssea lateral e condral medial dos côndilos femorais posteriores (grupo C), este estudo encontrou resultados relevantes para a diferença de aferições médias de 8,56. Desta forma, é necessária atenção ao perfil condral dos côndilos femorais posteriores para o planejamento rotacional do componente femoral quando usado o guia de apoio condilar. Ressalta-se que, no desgaste condral posterior lateral, mais prevalente em joelhos com sobrecarga em valgo, e que se assemelha à medida C deste estudo, os presentes achados podem ser de grande valia. Nesse cenário, a divergência média aproximada seria de 5° adicionais de rotação externa em relação ao valor empírico universal de 3°. Este fato acarretaria em um posicionamento do componente femoral em rotação interna, o que contribuiria para o aumento do conflito patelofemoral lateral e elevaria a tensão retinacular e a inclinação patelar, condições favorecedoras da síndrome patelofemoral pós-artroplastia. 11 12 13 14 A resultante cinemática não fisiológica deste joelho acarreta potencial para piores desfechos clínicos e dor anterior no pós-operatório. 3 4 5

Nam et al. 15 evidenciaram que a espessura média da cartilagem do côndilo femoral posterior lateral é maior do que a espessura da cartilagem do côndilo femoral posterior medial, com diferença média de 0,4mm, o que pode justificar a diferença significativa dos valores angulares encontrados entre as diferentes formas de medida do ECP analisadas. Além disso, a desigualdade da espessura condral dos côndilos femorais na osteoartrite do joelho pode variar de acordo com os desvios de eixo do membro e contribuir como fator causal para o erro na determinação do ECP. 16 Tashiro et al. 17 e Yang et al. 18 , ao compararem a espessura condral no aspecto posterior dos côndilos lateral e medial, demonstraram que um afilamento de apenas 2 mm da cartilagem no côndilo lateral pode gerar divergências de aproximadamente 2° no alinhamento rotacional do implante femoral.

Os resultados encontrados neste estudo dão embasamento adicional à recomendação para o uso de mais de um método de aferição do posicionamento rotacional do componente femoral, de forma a reduzir os potenciais erros de alinhamento devido a variações anatômicas – além de potenciais diferenças interobservador, ao delimitar os parâmetros anatômicos usados como referência. 19 20

Este estudo apresenta algumas limitações. Membros em valgo podem apresentar hipoplasia do côndilo femoral lateral, e não foram estudados os eixos mecânicos dos membros inferiores avaliados, o que pode ter introduzido um viés. Outra limitação é a de que o grupo da população brasileira estudada, em termos de número e idade, pode não refletir anatomicamente a totalidade da população devido à grande miscigenação étnica encontrada no Brasil.

## Conclusão

Considerando referenciais posteriores exclusivamente condrais ou exclusivamente ósseos, o ETE na população brasileira estudada apresenta aproximadamente 5° de rotação externa em relação ao ECP. A aplicação empírica de 3° de rotação externa no componente femoral, portanto, pode ser insuficiente para o alinhamento rotacional adequado na população brasileira, o que pode ser ainda mais pronunciado no geno valgo. Assim, destaca-se a importância de considerar a necessidade de técnicas individualizadas para o posicionamento rotacional ótimo do componente femoral, de forma a obter melhor acurácia.
